# A Scientometric Study on Industrial Effluent and Sludge Toxicity

**DOI:** 10.3390/toxics9080176

**Published:** 2021-07-28

**Authors:** Amid Mostafaie, Diogo N. Cardoso, Mohammadreza Kamali, Susana Loureiro

**Affiliations:** 1 Department of Biology and CESAM, Campus Universitário de Santiago, University of Aveiro, 3810-193 Aveiro, Portugal; amid.mostafaie@ua.pt (A.M.); sloureiro@ua.pt (S.L.); 2 Process and Environmental Technology Lab, Department of Chemical Engineering, KU Leuven, J. De Nayerlaan 5, 2860 Sint-Katelijne-Waver, Belgium; mohammadreza.kamali@kuleuven.be

**Keywords:** toxicology, wastewater, sludge, industry, scientometry

## Abstract

The growth of industrialization has led to an increase in the production of highly contaminated wastewater. Industrial wastewater contains highly complex compounds varying in characteristics and required to be treated before its discharge into a water medium from various industries. However, the efficiency of the treated wastewater from the toxicity reduction perspective is unclear. In order to overcome this barrier, toxicity assessment of the industrial wastewater before and after treatment is crucial. Thus, in this study, a scientometric analysis has been performed on the toxicity assessment of industrial wastewater and sludges, which have been reported in the literature. Web of Science (WoS) core collection database has been considered the main database to execute this analysis. Via the search of pre-researched keywords, a total number of 1038 documents were collected, which have been published from 1951 to 2020. Via CiteSpace software and WoS analyser, these documents went under analysis regarding some of the scientometry criteria, and the detailed results obtained are provided in this study. The total number of published documents on this topic is relatively low during such a long period of time. In conclusion, the need for more detailed contributions among the scientific and industrial communities has been felt.

## 1. Introduction

Industries such as pulp and paper, textile, cement, oil, leather, paint, food, among others, normally produce a massive amount of sludge and effluents [1,2,3,4]. Such wastes generally contain several environmental contaminants, which may affect environmental and human health [5,6]. Such effluents and sludges containing high levels of chemical oxygen demand (COD), biochemical oxygen demand (BOD), and the presence of recalcitrant environmental pollutants such as adsorbable organic halides (AOX), which are highly toxic for the environment [1,3,7,8,9].

The growth of industrialization and the discharges of the produced industrial effluents are some of the main sources of air, land, and water pollution [10,11]. Thus, environmental risk assessment procedures play a major role in evaluating the effects of the discharged industrial effluents on aquatic organisms, which are highly at risk due to discharge levels that may represent high pollution levels of natural resources [2]. The use of ecotoxicology assays for monitoring the efficiency of the treatment technologies and detoxification of industrial wastewaters has been applied in many studies [2,12]. However, there is still a lack of information on the toxicity characteristics of various industrial effluents.

Dealing with such high toxic effluents has been a subject of various studies worldwide. However, the number of reports in the literature for an effective application of novel and efficient methods to deal with this problem in real-scale applications are rare due to existing barriers such as the treatment costs and the need for high-tech equipment [1,2,7]. Due to this fact, the toxicity of the effluents released from industrial activities has continued to be a significant problem worldwide, especially in developing countries [13,14]. In this situation, in-depth knowledge of the industrial effluents’ real effects on the surrounding environment may help industries and scientific communities to develop a more efficient and cost-effective solution to deal with industrial effluents and sludge [9,15].

Scientometry is considered as a highly beneficial tool that measures and analyses scientific literature, aiming at investigating the history of science in a specific field and monitoring the active bodies worldwide, as well as the most important issues (as keywords) to focus on for future studies [16,17,18]. Hence, scientometric analysis can demonstrate the efforts performed in any specific field and mark existing barriers, milestones, and trends in the study field.

Despite the high-quality studies published in the literature, to the best of our knowledge, there are no scientometric studies performed regarding the ecotoxicological assessments of the real industrial effluents so far. Hence, this study aimed at providing scientometric aspects of industrial effluents toxicity followed by a critical discussion on the results and the aspects to be focused on for the future in order to diminish the drawbacks attributed to the production and release of highly polluted industrial effluents and sludge on the receiving ecosystems.

## 2. Materials and Methods

**Figure 1:**
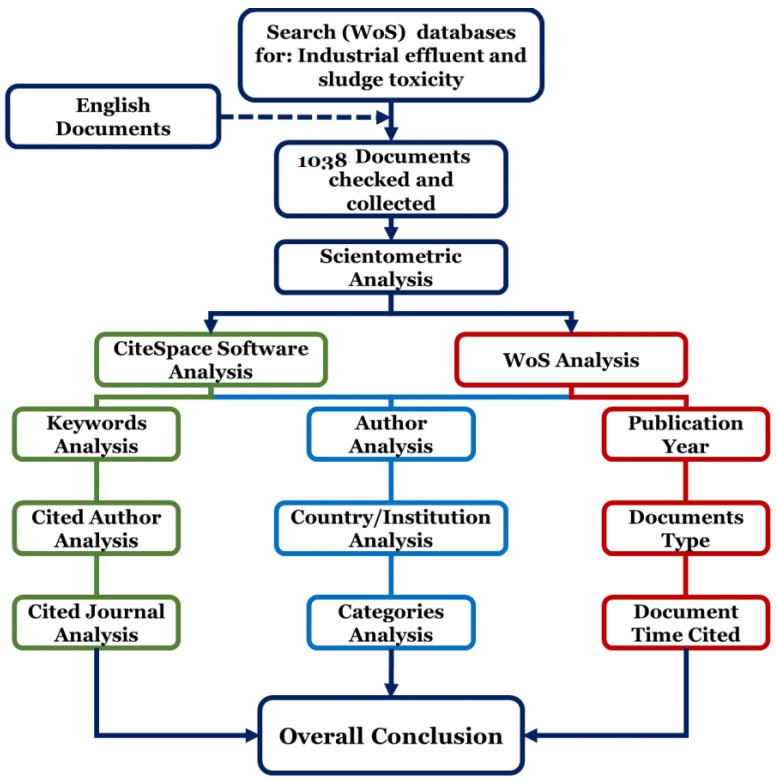
Research design used in the scientometric study performed for industrial effluent and sludge toxicity. To initiate this analysis’s execution, primarily, the search details, such as the pre-analyzed keywords and the English language, were inserted in the advanced search mode of WoS (shown with dark blue). Then, the obtained articles were separately analyzed via CiteSpace software (show in green), WoS analysis (shown in red), and the joint analysis of both analyzers (shown in light blue) regarding the scientometric criteria. Thereafter, conclusions were drawn based on the obtained results.

This study focuses on a scientometric analysis of industrial effluent and sludge toxicity to visualize and analyse the available documents in this regard. All studied documents have been obtained from the Web of Science (WoS) core collection platform (© 2020 Clarivate Analytics), which also covered most Institute for Scientific Information (ISI) publications from various core journals and is the universal database for ISI publications [19,20]. Hence, 1038 documents collected from WoS were analysed with CiteSpace program (Visualizing Patterns and Trends in Scientific Literature) and also WoS analysis (provided by (© 2020 Clarivate Analytics). The following keywords have been searched to assess all publications in this regard: * indust * or mill * or * factory * and effluent * or waste water * or wastewater * or waste * or sludge or landfill * or leachate * or release * or fate * or discharge or influent * and * toxic * or mutagen * or detoxif * or lethal. A fuzzy search has been applied with “*” and we did not apply any predefined specific duration of time to find out all publications. No limited duration was considered since the number of retrieved documents with the applied keywords was scarce. In this way, the utilized database was equipped with the opportunity to provide all the documents published at any time in the past. The performed research design is presented in Figure 1. There are four parameters for the scientometric analysis;

(1) centrality: this parameter represents the significance of any data. Whenever any types of data, such as a keyword or author name, are located in the centre of the figure, that data is of high importance;
(2) burst: this parameter is a tool to measure the frequency of the appearance of any type of data, whether a keyword or an author, over a specific and short duration of time;
(3) sigma: this is an integrated measurement in CiteSpace software, which demonstrates the combination of both the citation and the burst of any type of data, whether a keyword or an author’s name;
(4) clustering: this is a measurement of the data by categorizing the information based on the similarly utilized keywords. According to the size of the category, each cluster gains a size. The largest cluster containing the most homogenous data is demonstrated by #0 [21].

## 3. Results

Following the research design presented in Figure 1, the total number of 1038 publications was gathered. All these documents are in English from the year range of 1951 to 2020. Furthermore, the gathered documents were taken under analysis regarding some specific scientometric parameters as “the publication year”, “document type”, “keywords co-occurrence”, “authors”, “country and institute”, “cited authors”, “cited journals”, “categories”, and “time cited documents”. In the following sections, these parameters will be further discussed.

### 3.1. Publication Years Analysis

**Figure 2:**
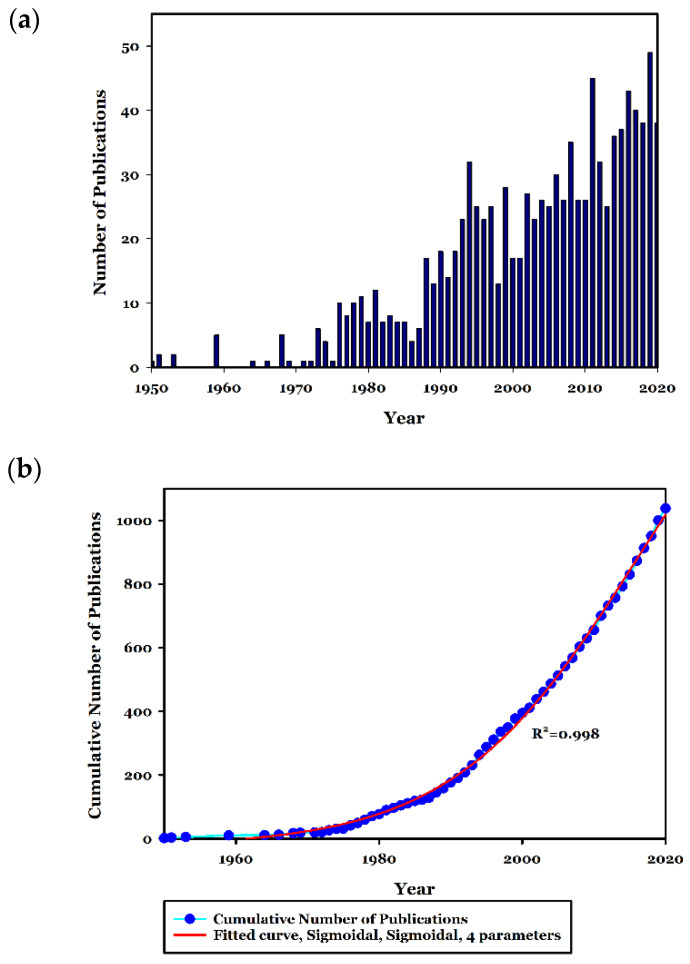
The number of scientific documents published on industrial effluent and sludge toxicity performed on industrial wastewater from 1950 to 2020 (**a**). Fitted curve and cumulative number of publication in the adopted duration (**b**).

All obtained documents from 1951 to 2020 are shown in Figure 2, where the number of publications per year can be observed, and the cumulative publications number pattern. Table 1 represents the number of publications in the last decade, which includes more than 39% of all publications. The pattern of the cumulative number of publications with the fitted curve of sigmoidal four parameters (Rsqr = 0.9987) represents the sigmoidal growth of the number of publications over the adopted period.

### 3.2. Documents Types

**Figure 3:**
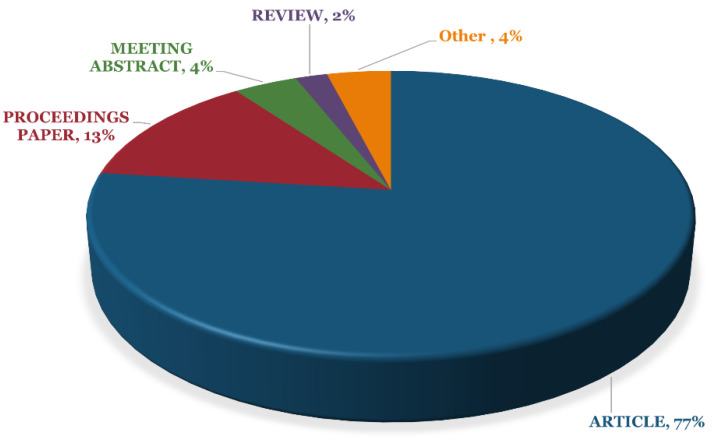
The obtained results demonstrating the types of published scientific documents on toxicity analysis on industrial wastewater during the adopted period (1950–2020) gathered from WoS.

The results obtained from the search of the keywords mentioned above in WoS have been furthered analysed based on the type of published documents. Accordingly, the results indicate that most of the scientific documents in the field of toxicity analysis on industrial wastewater have been published as articles with an overall portion of 77%. Furthermore, proceeding papers (with a portion of 13%) and meeting abstracts (with the portion of 4%) were observed to include the highest types of published documents in this field (Figure 3).

### 3.3. Keyword Analysis

**Figure 4:**
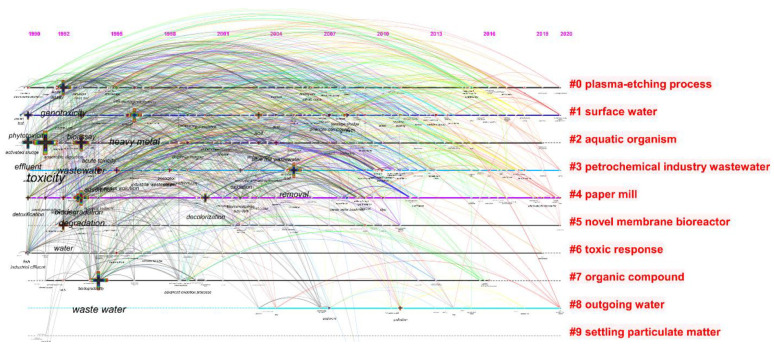
The timeline of the keywords appears in the scientific documents published in the field of industrial effluent and sludge toxicity from 1950 to 2020 via the utilization of CiteSpace software.

The chosen documents in the field of toxicity analysis of industrial effluents in WoS were further examined based on the most displayed keywords via CiteSpace software. The results achieved from this analysis demonstrate the keyword “toxicity” with the frequency of 148 has appeared the most. Moreover, according to the timeline of the appeared keywords, Figure 4 shows that the keyword “toxicity” has appeared between the years 1990 and 1992 for the first time. Furthermore, the keywords “waste water” and “wastewater” have appeared more frequently compared to other keywords with the frequencies of 100 and 95, respectively. As the software cannot distinguish the difference in writing, the keywords “wastewater” and “waste water” have been considered two different keywords. The keyword “heavy metal” with the respective frequency of 83 has been categorized as the fourth keyword appearing the most.

Moreover, the keywords have been analysed based on the parameter burst during the adopted period from 1950 to 2020. Accordingly, the keywords “water”, “effluent”, and “heavy metal” with the amount of burst of 10.92, 10.34, and 6.29, respectively, contained the highest burst. The detailed characteristics of the appeared keywords are represented in Table 2.

As mentioned above, the timeline in which the keywords have appeared has been obtained from the keyword analysis from CiteSpace software. The timeline of keywords has been divided into nine clusters. The cluster entitled “Plasma-etching process” (cluster#0) and “surface water” (cluster#1) were distinguished to be the largest clusters obtained from this analysis.

**Figure 5:**
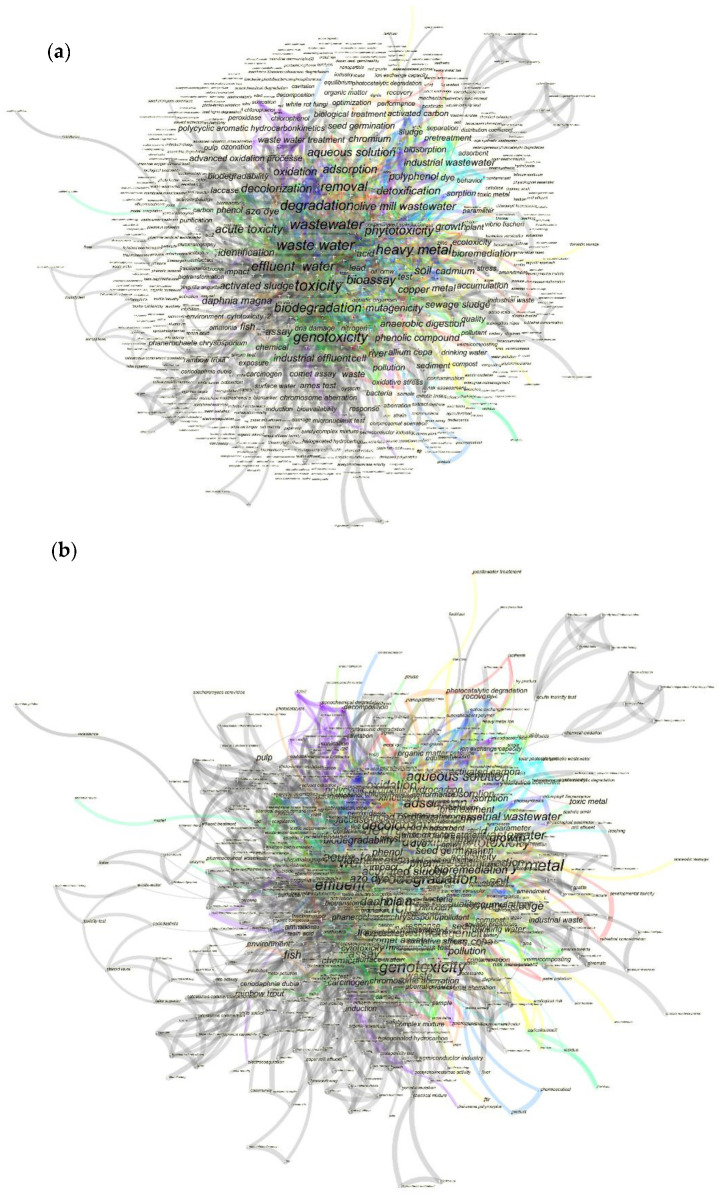
The analysis regarding the appeared keywords in the scientific documents published on the industrial effluent and sludge toxicity analysis from 1950 to 2020 via CiteSpace software. Figure (**a**) represents the keywords’ analysis without the centrality for better visualization, and (**b**) demonstrates the same figure with the respective centrality.

In Figure 5 we can observe the visualization of all the obtained keywords during the mentioned time with minimized overlaps (a) and without any changes in centrality (b).

### 3.4. Authors’ Analysis

**Figure 6:**
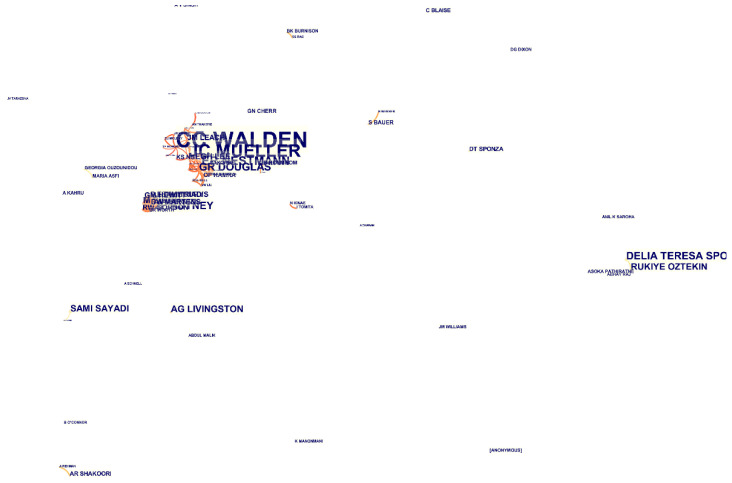
The analysis regarding the authors who have contributed to the field of industrial effluent and sludge toxicity during the adopted period achieved from the utilization of CiteSpace software.

The obtained documents from the search of the keywords mentioned above in WoS were furthered inserted in CiteSpace software to be analysed regarding the authors who contributed to the field of toxicity analysis of industrial effluents. Figure 6 and Table 3 represent the results achieved from this analysis. As it can be observed, the authors “Walden. CC” (frequency = 16 and burst = 9.25), “Mueller. JC” (frequency = 13 and burst = 7.5), and “Nestmann. ER” (frequency = 9 and burst = 5.17) have contributed the most in this field. In this analysis, the nodes represent the authors, while the links demonstrate the contribution among authors. As shown in Figure 6, no significant connections have been formed among the authors who are accounted to be contributing to the field of toxicity analysis on the industrial wastewaters except very few groups.

### 3.5. Contributed Countries/Institutions

**Figure 7:**
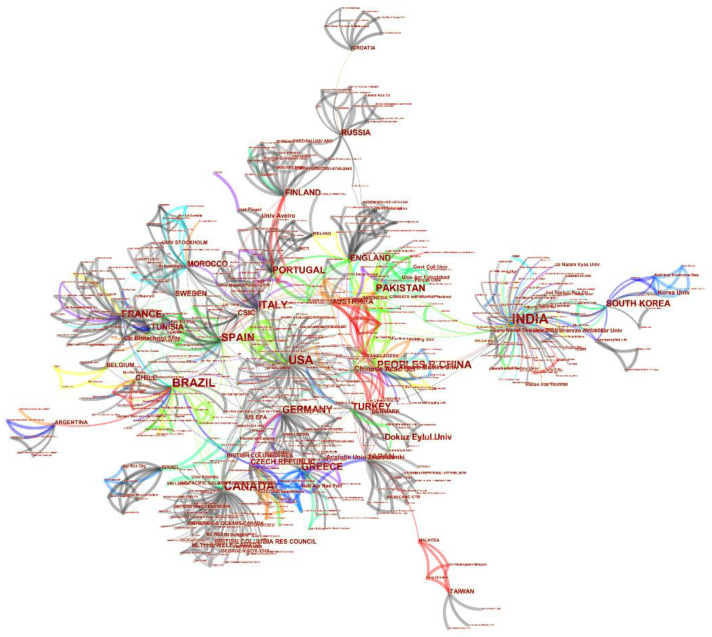
The analysis regarding the contributing countries and institutions in the field of industrial effluent and sludge toxicity from 1951 to 2020. This analysis has been achieved via the utilization of CiteSpace software.

**Figure 8:**
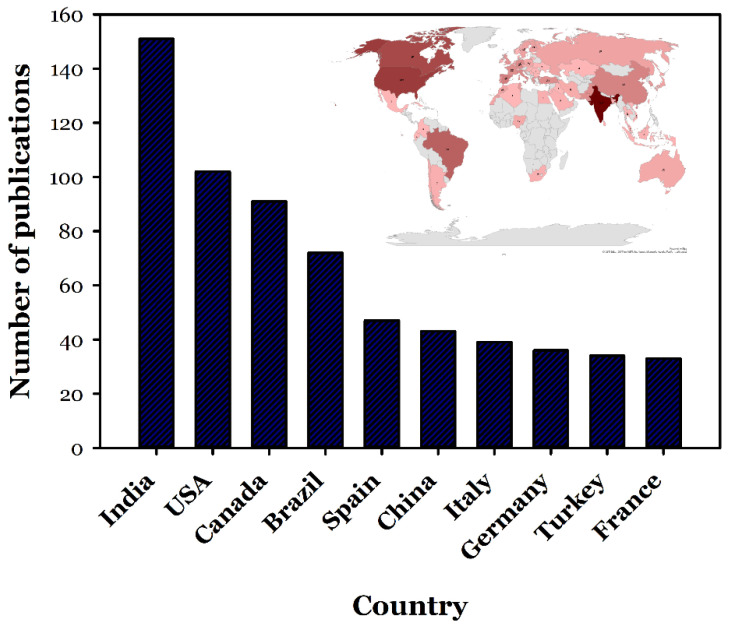
Countries that most contribute to the field of the industrial effluent and sludge toxicity analysis with their respective number of published documents over the adopted duration from 1951 to 2020. The most contributing countries in this field have been presented with a darker color in the map, and the variation in the utilized color demonstrates the number of contributions provided by different countries.

The documents published in the toxicity analysis field on industrial effluents from 1951 to 2020 were further analysed based on the contributed countries and institutions. This analysis has been performed via CiteSpace software utilization, and the respective results are shown in Figure 7 and Figure 8, and Table 4. According to the achieved results, India, the USA, and Canada are accounted as the most contributing countries in this field with the numbers of publications of 151 (with an overall portion of 14.55%), 102 (with an overall portion of 9.83%), and 91 (with an overall portion of 8.77%), respectively. Although India has the first rank of studies in this regard, the population ratio to the number of executed research in Table 4 shows that Canada has the most valuable rank with 0.41 million people per study, and the lowest rank in this table is for China with 33.47 million people per study. In this analysis, and based on Figure 7, the nodes represent the countries, and the links stand for the co-contribution of countries in this field. Moreover, in this analysis, the institutions with the highest level of contribution in this field have also been demonstrated in Figure 7. The utilized fonts demonstrating the countries and the institutions represent their respective number of contributions in this field. The bigger the utilized font, the highest amount of contribution of the respective country/institution.

### 3.6. Cited Authors and Organizations Analysis

**Figure 9:**
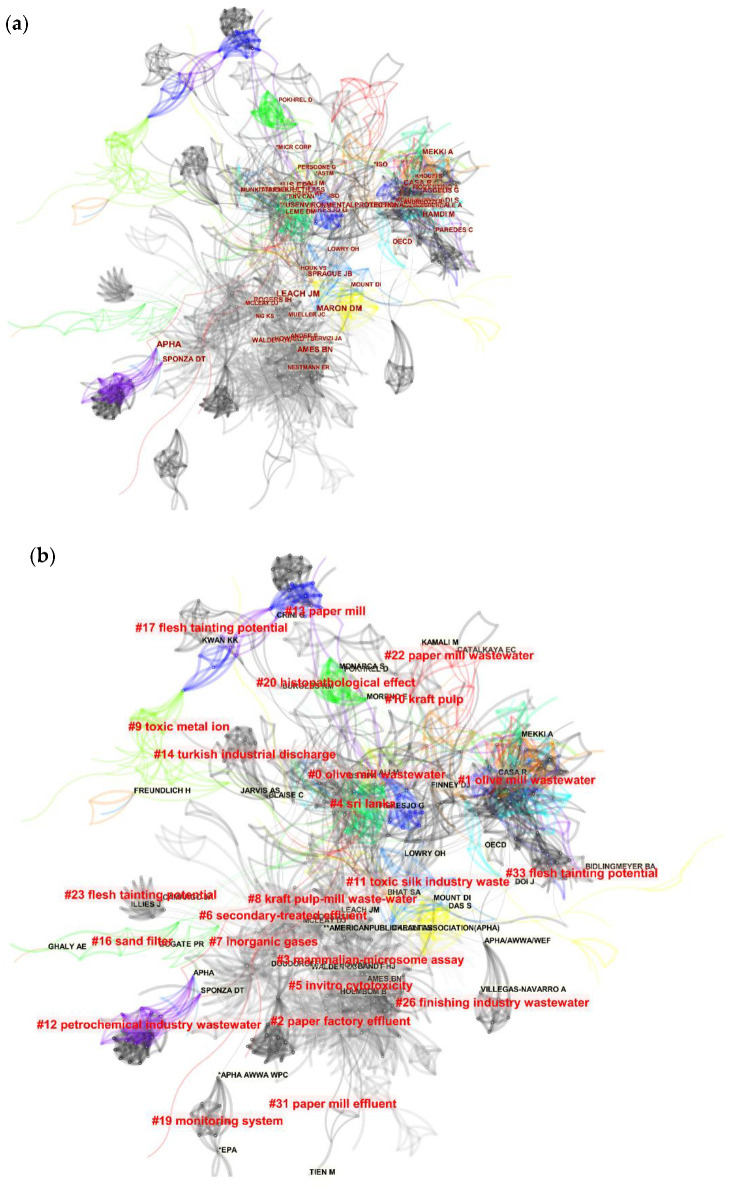
The obtained results from the analysis of the cited authors and organizations contributing to the field of industrial effluent and sludge toxicity from 1951 to 2020. Both illustrations (**a**,**b**) have been achieved via the utilization of CiteSpace software. Figure (**a**) represents the cited authors in this field, while figure (**b**) illustrates the clustering applied to the most cited authors.

The list of authors and organizations of the chosen documents were examined regarding the number of citations they received during this period. Accordingly, the organizations and authors “APHA” (frequency = 58 and burst = 15.2), “Leach JM” (frequency = 42 and burst = 18.21), and “US EPA” (frequency = 39 and burst = 11.81) were indicated to have been receiving the highest number of citations. This analysis has been performed via the utilization of CiteSpace software. The respective achieved results in this regard are demonstrated in Figure 9a. Furthermore, Figure 9b illustrates the clustering of keywords based on the cited authors and organizations. As can be observed, “olive mill wastewater” with 150 members is the largest cluster (cluster#0). Moreover, the top five cited authors and organizations with their respective scientometric characteristics are represented in Table 5.

### 3.7. Cited Journals Analysis

**Figure 10:**
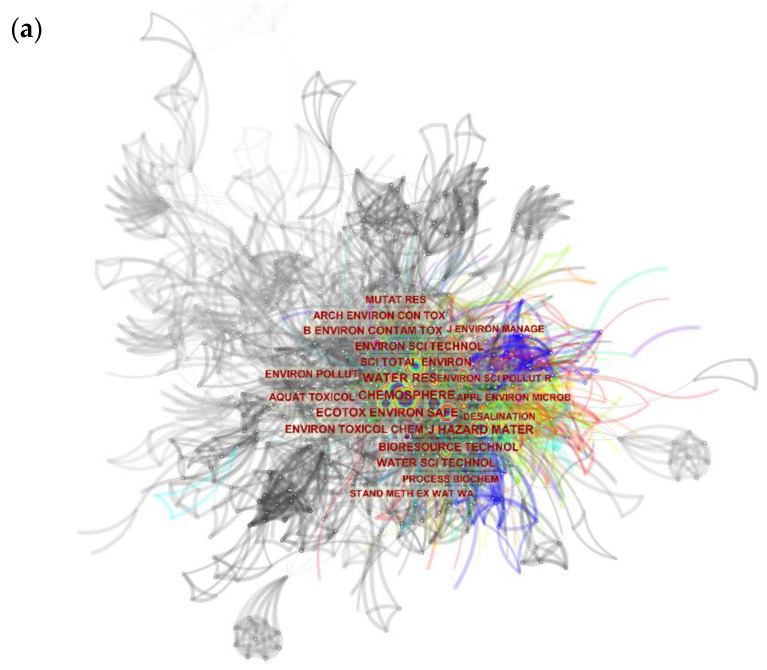

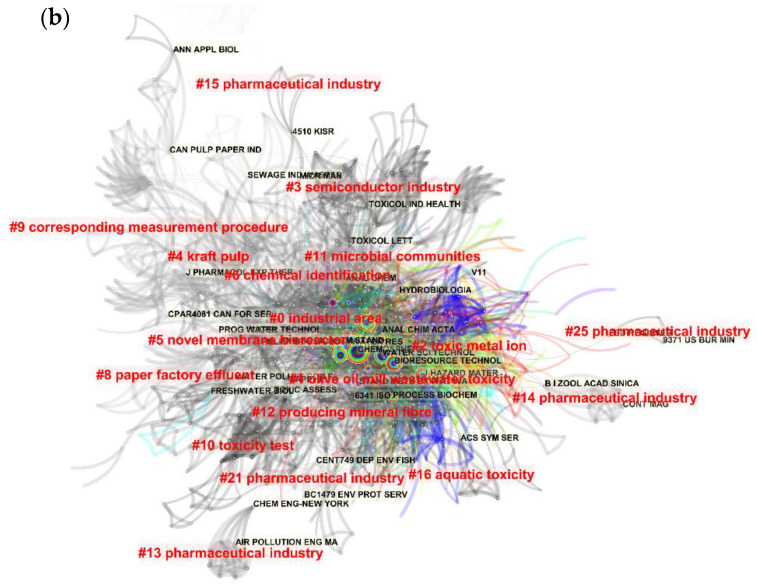
The results were achieved to analyze the most cited journals contributing to the field of industrial effluent and sludge toxicity from 1951 to 2020(the visualization of the cited journals (**a**) and the visualization of clustering of the cited journals (**b**)).

The obtained scientific documents on the industrial effluent and sludge toxicity analysis from 1951 to 2020 were further examined based on the most cited journals. Figure 10 and Table 6 demonstrate the obtained results in this regard via the utilization of CiteSpace software. Based on the achieved results, the journals “Chemosphere” (frequency = 409 and burst = 0.55), “Water Research” (frequency = 406 and burst = 5.89), “Ecotoxicology and Environmental Safety” (frequency = 274 and burst = 2.41) and “Journal of hazardous material” (frequency = 264 and burst = 10.82) were identified to be the journals with the highest received citations. Moreover, as can be observed from Figure 10b, the keywords clustering based on this analysis demonstrates 25 clusters with “industrial area” as the largest cluster (#0) with 208 members.

### 3.8. Categories Analysis

**Figure 11:**
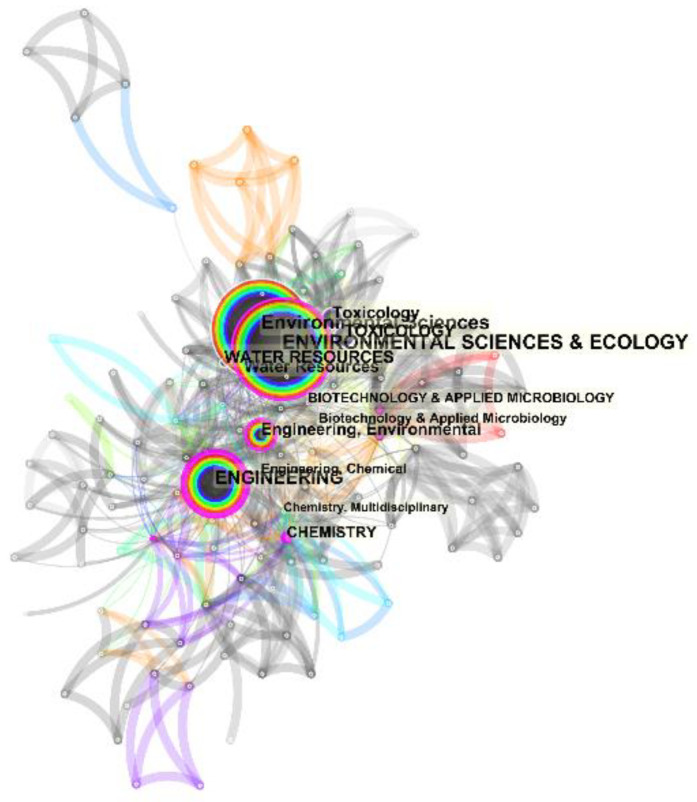
The analysis was performed on the categories in which the documents on industrial effluent and sludge toxicity from 1951 to 2018 were divided into. This analysis has been executed via the utilization of CiteSpace software.

The obtained documents from the search of the keywords mentioned above in the industrial effluent and sludge toxicity from 1951 to 2020 were furthered analysed based on the categories they were divided into. This analysis has been performed via the utilization of CiteSpace software, and the respective result is presented in Figure 11 and Table 7. As can be observed, most of the considered documents were in the field of “environmental sciences & ecology” with a record count of 584. The second and third categories with the highest number of record counts were observed to be “Environmental Sciences” and “Engineering” with the respective record counts of 577 and 296, respectively.

### 3.9. Time-Cited Documents

The documents considered in this study on the industrial effluent and sludge toxicity from 1951 to 2020 were analysed based on the number of citations they received during the mentioned period. This result has been obtained by the WoS analysis tool. The titles of the top 10 documents with the highest received citations in the bank of WoS have been represented in Table 8. Their respective year when they were published, and their corresponding number of received citations have also been included in Table 8.

## 4. Discussion

The first aim of this study was to execute a comprehensive scientometric approach regarding the research performed in the field of industrial effluent and sludge toxicity. The results indicate that only 1038 bibliographic documents have been published between 1951 to 2020, which is quite a low number for a relatively long time, especially compared to the other scientometric studies [16,19]. This may reveal the urgent need for more research and effort in this area to have a scientific-based conclusion about the toxicity of the effluents and sludge from various industrial origins. The number of publications (Figure 2) demonstrates a sigmoidal trend indicating that the number of publications is even in a declining mode. In this regard, perhaps the public support, mainly by providing financial requirements and the development of the related standards and regulations, may accelerate scientific research in this field. Moreover, fluctuations in the number of publications in this area may indicate that this field’s scientific progress has not followed a steady state. In this regard, perhaps there is a lack of effective cooperation of the industry with the scientific community to explore the toxicity of the produced effluents and the effectiveness of the treatments used. So to promote the cooperation of the industry with the scientific community, some actions to fill existing gaps should be attained: (1) existence of effective regulations, which may obligate the industries for such activities; or (2) the stringent environmental standards on the discharged effluents quality, which may bring the need for improvement of the treatment systems located in the industries, seems to be essential [11,22]. In this regard, and despite the efforts of international organizations, such as the environmental protection agency (EPA), for the development of relevant regulations and directives, there are currently no obligations for industries to perform a direct toxicity evaluation on their discharged industrial effluents [31]. Also, it must be stated that although the efficient treatment facilities can reduce the load of harmful chemical compounds such as AOX, BOD, COD, phenolic compounds, among others [9], there is still no guarantee for the reduction in toxicity of the industrial effluents even after the treatment applied [32]. Some measures that are normally adopted for the degradation or removal of specific contaminants may cause some toxic effects, for instance, by releasing chemical compounds to the content of effluents, especially when chemical treatment techniques are used. Besides, the effects of complex mixtures are disregarded when solely chemical analysis is used to establish levels of concern. As represented in Figure 3, more than 77% of all documents published in this field are categorized as “articles” which may reveal the tendency of the researchers to publish their findings in indexed journals rather than as conference papers and books. Moreover, according to Table 6, the journals in the field of environmental science and engineering, like “Chemosphere” and “Water research”, have been active in the publication of the findings in this scientific field, but those which have specifically published ecotoxicological findings are rare among the top publishers in this area.

The keywords timeline illustrated in Figure 4 shows that between 1990 and 2000 a number of critical keywords on the toxicity of industrial effluent and sludge have appeared in the scientific documents. Keywords such as “toxicity”, “wastewater”, and “heavy metal” might have been used previously; however, they have been mainly highlighted and gained more attention in the period mentioned above. As represented in Figure 4, the clusters entitled as “plasma-etching process” (with a waste gas mixture of chlorinated hydrocarbons and inorganic by-products [33]), “pulp and paper mill” (with a high amount of sodium hydroxide, chlorinated organic compounds, low BOD, and high COD [34]), “olive oil mill” (with a high amount of high organic load, phytotoxic and antibacterial phenolic substance, [35]), and “petrochemical”(with polycyclic aromatic hydrocarbon [36]) in their discharges, are categorized as the most toxic industrial effluents for the receiving environments. Other industries, such as textile mills, normally produce highly concentrated toxic wastewaters laden with dyes, low BOD, and high COD [37]. The third biggest cluster represented as #2, entitled “aquatic organisms”, emphasizes the importance of the execution of toxicity evaluation testing via aquatic organisms, such as *D. magna,* which appeared in 1999. However, this test organism has not been presented in big fonts which demonstrates that this organism has not gained sufficient attention. Moreover, the fifth-largest cluster represented as #5 is called “novel membrane bioreactors”, which indicates that this technique of wastewater treatment has been applied the most. By a closer look at this cluster, the main keyword is “biodegradation”, represented with a big plus symbol, and which complies with the fouling problem of this treatment method and also it can be distinguished as a barrier [19]. Thus, more novel treatment techniques can be considered essential. The trend keywords represented in this figure via a plus symbol are “anaerobic digestion”, “phytotoxicity”, “degradation”, “metal”, and “olive oil”, respectively, listed from the most to the least important. The main top four trends appeared before 2000 and only the least significant trend, “olive oil”, appeared in 2005. After 2005, no significant trend keyword has been observed. Moreover, as shown in this figure, after 2010, only a few keywords have been entered in the scientific literature in this field. This may demonstrate that there is still a lack of in-depth studies, including keywords such as “nanotoxicology” as an emerging global issue caused by the application of nanomaterials for wastewater treatment [38].

According to Figure 6, there are just a few collaborating authors in the field of toxicity assessment of industrial effluents. As can be observed, no extensive collaboration links have been formed, even between some of the most active authors in this field, such as Walden.CC, Mueller.JC, and Douglas.GR (represented with the biggest font as their contribution has been more). Although some collaborations between some countries and institutes can be identified from this figure, due to their cooperation on international projects, a low degree of collaborations is observed among the individual researchers. Figure 7 and Figure 8 represent the share of the countries and institutes in this field. As can be observed, India is the most active country in publishing bibliographic records on industrial wastewater’s toxicity. Besides the government’s strict regulations, the amount of effluents produced in India and subsequent environmental drawbacks can be the most important reasons for India’s position in this regard [39]. The USA, Canada, Brazil, and Spain are the most active countries in this field after India. Also, China, which is accounted as one of the most industrialized countries with high contributions in the scientific document production in the field of wastewater treatment (e.g., in applying MBR bioreactor [19]), has no significant contribution in the field of the industrial wastewater toxicity. The progress in scientific research in this field needs pressure from stakeholders, customers, and communities [40] rather than the degree of the country’s development. As stated before, the most active country in this field is India, which, as a developing country, possesses highly toxic industries.

The most cited collected documents represented in Table 8 show no highly cited publications in the last decade in this area. Moreover, the top rank publication in this regard was published in 2001 as a review article on pulp and paper mill effluent [1]. The latest highly cited publication represented in Table 8 was published in 2014 regarding the toxicity assessment of a pyrolyzed biochar produced from the sludge of treated pulp and paper mill wastewater [26]. The top two highly cited papers represent the importance of pulp and paper mill and olive oil mill wastewaters from the toxicity perspective, which requires considerable capital and operating capital to treat the produced effluents. In addition to the mentioned industries, there are several industries, such as food, paint, and textile, which are not highlighted in the (1038) collected bibliographic records. It means that although these industries produce highly toxic wastewaters and effluents, there are not enough reports published in the literature investigating their toxicity effects. However, there is no clear information regarding the reduction of the toxicity of the industrial effluents after the treatments as different test organisms have a different response in toxicity evaluation [32]. Also, each industry has its own treatment methods, which should be evaluated with different tests to provide clear information regarding their wastewater toxicity after treatment.

## 5. Conclusions

Industrial effluents and sludges are among the most critical sources of highly polluted and contaminated residues and can cause many environmental and ecological issues upon discharge to the environment. A comprehensive scientometric analysis has been carried out in this study to provide a clear understanding of the scientific efforts regarding the analysis of industrial effluents’ toxicity assessment. India and the USA accounted as the leading countries contributing the most in this regard by evaluating and analyzing the industrial effluent to avoid environmental contamination. These kinds of studies are accounted for as the result of the function of economic situations and policies. Therefore, the absence of effective regulations and standards regarding these types of effluents’ toxicity can be felt globally. However, it is inevitable that even with strict regulations, industries might not cooperate as effectively as they should due to high capital and monitoring costs. Thus, a need for more cooperation among the scientific and industrial bodies is felt to overcome the obstacle of monitoring and controlling effluents’ toxicity level before their discharge into the water media.

## Figures and Tables

**Table 1:** The number of published documents in the last 10 years and the respective percentage of the publications in each year.

Year	Publication (no.)	Portion (%)
2020	38	3.66%
2019	49	4.72%
2018	38	3.66%
2017	40	3.85%
2016	43	4.14%
2015	37	3.57%
2014	36	3.47%
2013	25	2.41%
2012	32	3.08%
2011	45	4.34%
2010	26	2.51%

**Table 2:** The keywords’ characteristics appeared in the scientific documents published in the industrial effluent and sludge toxicity analysis regarding their scientometric specifications (frequency and burst). These results have been gained from the analysis performed via the utilization of CiteSpace software. The symbol “-” utilized represents that the specific keyword does not have any value corresponding to a burst in any duration of time.

Rating	Keyword	Frequency	Burst
1	toxicity	148	-
2	waste water	100	-
3	wastewater	95	-
4	heavy metal	83	6.29
5	degradation	79	-
6	genotoxicity	76	-
7	effluent	69	10.34
8	removal	66	5.55
9	water	58	10.92
10	biodegradation	55	-
11	bioassay	54	-
12	phytotoxicity	45	-
13	decolorization	41	-
14	adsorption	41	-
15	aqueous solution	36	4.85
16	soil	36	-
17	acute toxicity	35	-
18	olive mill wastewater	33	5.79
19	detoxification	30	-
20	oxidation	29	4.89

**Table 3:** The characteristics (frequency, burst, and record count) of the authors contribute to industrial effluent and sludge toxicity based on the achieved documents during the adopted duration from 1950 to 2020 obtained from CiteSpace. Accordingly, the number of documents published by each of the authors in this field and during this period has been gathered from WoS analysis. They are represented as “record counts”.

Rating	Author	Frequency	Burst	Record Count (No.)
1	CC Walden	16	9.25	16
2	JC Mueller	13	7.5	13
3	ER Nestmann	9	5.17	9
4	GR Douglas	8	4.59	8
5	Delia Teresa Sponza	8	4.46	13
6	AG Livingston	7	3.93	7
7	JP Kutney	7	4.37	7
8	A Oilkari	6	0.55	7
9	Sami Sayadi	6	3.87	9
10	JA Servizi	6	3.05	6

**Table 4:** The analysis regarding the most contributing countries in the field of industrial effluent and sludge toxicity during the period of 1951 to 2020 via utilization of CiteSpace software. As can be observed in this table, India and the USA, with the total portion of 14.55% and 9.83%, respectively, have contributed the most in publishing scientific documents in the field of toxicity analysis of industrial effluents from the numeric perspective. Moreover, by comparing the corresponding population ratio, Canada has shown a more productive behavior in publishing the scientometric publications in this regard.

Rating	Country	Record Count (No.)	Portion (%)	Population Ratio to the Amount of Executed Research (Million People/Study)
1	India	151	14.55%	9.18
2	USA	102	9.83%	3.24
3	Canada	91	8.77%	0.42
4	Brazil	72	6.94%	2.95
5	Spain	47	4.53%	0.99
6	China	43	4.14%	33.47
7	Italy	39	3.76%	1.55
8	Germany	36	3.47%	2.33
9	Turkey	34	3.28%	2.49
10	France	33	3.18%	1.98

**Table 5:** The list of the top 10 cited authors and organizations contributing to the field of industrial effluent and sludge toxicity analysis from 1951 to 2020 was performed via CiteSpace software. The authors and organizations’ respective scientometric characteristics have also been presented, with their frequency and burst parameters.

Rating	Authors	Frequency	Burst
1	APHA	58	15.2
2	Leach JM	42	18.21
3	US EPA	39	11.81
4	Maron DM	34	7.19
5	Ames BN	33	11.61

**Table 6:** The top 10 highly cited journals in the field of industrial effluent and sludge toxicity from 1951 to 2020. Some of the mentioned journals do not possess a corresponding burst amount since they did not receive a high number of citations over a short period of time (represented with “-”).

Rating	Cited Journals	Frequency	Burst
1	Chemosphere	409	0.55
2	Water Research	406	5.89
3	Ecotoxicology and Environmental Safety	274	2.41
4	Journal of Hazardous Materials	264	10.82
5	Science of the Total Environment	244	-
6	Environmental Science & Technology	233	6.7
7	Bioresource Technology	226	2.37
8	Environmental Toxicology and Chemistry	207	2.89
9	Water Science & Technology	203	13.29
10	Bulletin of Environmental Contamination and Toxicology	203	1.74

**Table 7:** The top 10 categories in which the documents on the industrial effluent and sludge toxicity analysis from 1951 to 2018 were divided into. The results have been achieved by the CiteSpace software. The first two categories with the highest corresponding frequencies have not received a value regarding their burst. These two categories were focused on this field more frequently and not specifically in a short duration of time, which is defined as a burst as the scientometric criterion.

Rating	Categories	Record Count (No.)	Burst	Centrality	Sigma
1	Environmental Sciences and Ecology	584	1.9	0.16	1.33
2	Environmental Sciences	577	2.11	0.09	1.2
3	Engineering	296	1.31	0.4	1.55
4	Engineering, Environmental	210	1.39	0.11	1.15
5	Toxicology	202	2.49	0.06	1.15
6	Water Resources	169	0.54	0.05	1.03
7	Chemistry	92	1.07	0.23	1.25
8	Engineering, Chemical	81	5.23	0.02	1.12
9	Biotechnology & Applied Microbiology	76	3.96	0.06	1.24
10	Chemistry, Multidisciplinary	53	0.49	0.04	1.02

**Table 8:** The titles of the top 10 documents in the field of industrial effluent and sludge toxicity with the highest number of received citations according to WoS analysis.

Rating	Title	Year	Time Cited (NO.)	Reference
1	Aquatic toxicity from pulp and paper mill effluents: a review.	2001	361	[1]
2	The genotoxicity of industrial-wastes and effluents.	1992	239	[22]
3	Bio-assay methods for the evaluation of acute toxicity of industrial wastes to fish.	1951	216	[23]
4	Critical review of literature on the toxicity of industrial wastes and their components to fish.	1953	197	[24]
5	Genotoxicity of industrial wastes and effluents.	1998	185	[25]
6	Risk analysis of pyrolyzed biochar made from paper-mill effluent treatment plant sludge and eco-toxicity of heavy metals.	2014	154	[26]
7	Environmental effects caused by olive-mill wastewaters: Toxicity comparison of low-molecular-weight phenol components.	2003	154	[27]
8	Toxicity evaluation of reactive dyestuffs, auxiliaries and selected effluents in textile finishing industry to luminescent bacteria *Vibrio fischeri.*	2002	149	[28]
9	Land spreading of olive mill wastewater: Effects on soil microbial activity and potential phytotoxicity.	2007	142	[29]
10	Reduction of phenol content and toxicity in olive oil mill waste waters with the ligninolytic fungus *Pleurotus ostreatus.*	1996	140	[30]

## Data Availability

The data presented in this study are available on request from the corresponding author.
